# Epidemiology and early predictors of Fabry nephropathy: evaluation of long-term outcomes from a national Fabry centre

**DOI:** 10.1007/s40620-024-02170-9

**Published:** 2024-12-19

**Authors:** Fahmida Mannan, Rajkumar Chinnadurai, Ryan Wiltshire, Jan Hansel, Karolina M. Stepien, Reena Sharma, Gisela Wilcox, Eamon McCarron, Philip A. Kalra, Ana Jovanovic

**Affiliations:** 1 The School of Medicine, Manchester Academic Health Sciences Centre, Manchester University, Manchester, UK; 2 Division of Cardiovascular Sciences, Faculty of Biology, Medicine and Health, Manchester University, Manchester, UK; 3 Adult Inherited Metabolic Diseases, Salford Care Organisation, Northern Care Alliance NHS Foundation Trust, Salford, Greater Manchester UK; 4 Department of Renal Medicine, Salford Care Organisation, Northern Care Alliance NHS Foundation Trust, Salford, Greater Manchester UK; 5 Division of Diabetes, Endocrinology and Gastroenterology, University of Manchester, Manchester, UK; 6 Division of Immunology, Immunity to Infection and Respiratory Medicine, The University of Manchester, Manchester, UK; 7 Acute Intensive Care Unit, Manchester University NHS Foundation Trust, Manchester, UK

**Keywords:** Albuminuria, eGFR, Enzyme replacement therapy, Fabry disease, Nephropathy

## Abstract

**Background:**

Fabry disease is a rare genetic lysosomal storage disorder, whereby the accumulation of sphingolipids consequently leads to kidney structural damage and dysfunction. We explored the epidemiology of chronic kidney disease (CKD) among patients with Fabry disease at a major UK referral centre in Greater Manchester serving over 7 million people, to inform early predictors of kidney disease and possible treatment planning.

**Methods:**

Data were sourced from the electronic records of registered participants from November 2020 to February 2022 of adults diagnosed with Fabry disease, with at least 1 year of follow-up. Four hundred and five participants (female = 223, male = 182) met the initial eligibility criteria. Our study focused on identifying factors linked to incident CKD, with 395 evaluable individuals undergoing outcome analysis over a median of 6.4 years.

**Results:**

Findings concluded that 60.5% of participants received disease-modifying treatments, 29.7% experienced non-fatal cardiovascular events, 3.3% developed end-stage kidney disease (ESKD), and 7.3% died. Men had higher use of disease modifying therapy, progression to ESKD requiring kidney replacement therapy, cardiovascular events, and mortality compared to women. Subgroup analysis over 9 years revealed that older age, cardiovascular history, renin–angiotensin–aldosterone system inhibitor use, and higher urine albumin-to-creatinine ratio (uACR) were predictors of faster estimated glomerular filtration rate (eGFR) decline and increased mortality. At baseline, 47.8% of 249 patients with uACR data had CKD, and 25.4% of the remaining individuals developed CKD during follow-up, associated with higher uACR and lower, albeit normal eGFR levels.

**Conclusion:**

Over 60% of Fabry disease patients are at lifetime risk of developing CKD, with a substantial risk of mortality, even with initially normal uACR and eGFR values.

**Graphical abstract:**

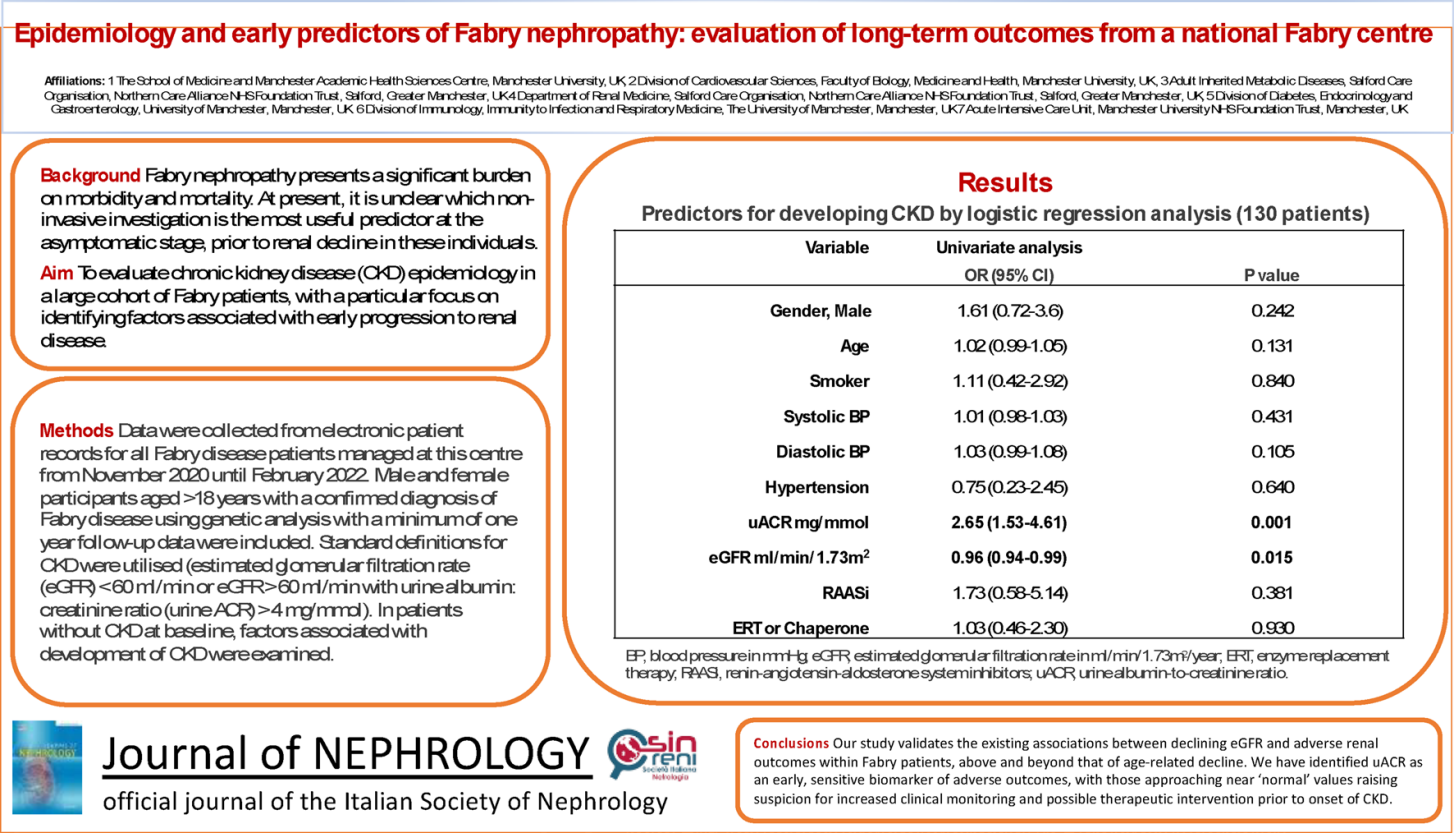

**Supplementary Information:**

The online version contains supplementary material available at 10.1007/s40620-024-02170-9.

## Introduction

Fabry disease (FD; OMIM 301500) is a rare, x-linked lysosomal storage disorder [1] that occurs due to mutations in the *GLA* gene, resulting in the absence or deficiency of α-galactosidase-A, causing tissue glycolipid accumulation and devastating kidney dysfunction [2–5].

Fabry disease encompasses two phenotypes: classical or non-classical (also known as late-onset/adult variant), respectively[1, 2]. Classical Fabry disease typically affects men in early life causing end-stage kidney disease (ESKD), stroke and hypertrophic cardiomyopathy [2, 4]. Individuals with late-onset Fabry disease and residual α-galactosidase-A activity usually (but not always) present from the third decade of life [1]. This variability of Fabry disease is additionally influenced by the degree and direction of X-chromosome inactivation in heterozygous females, alongside epigenetic factors including allele-specific DNA methylation at the *GLA* gene promoter site, again affecting disease onset and outcome [5].

The global prevalence of Fabry disease is heterogenous; it was previously estimated to be 1:117,000 male births [6]. However, analysis of *GLA* gene variants in exome sequencing data from 200,643 individuals from the UK Biobank revealed a heterogenous prevalence of 1 in 5732 for late-onset disease-causing variants, and 1 in 200,643 for classic Fabry disease variants [7]. Current disease modifying treatments for Fabry disease are limited to direct enzyme replacement therapy or oral chaperone therapy, with the latter serving to stabilise and improve the function of endogenously produced α-galactosidase-A [8].

The above demonstrates a need to identify predictive biomarkers prior to the onset of kidney damage to enable the initiation of timely and effective treatment.

## Objectives

We aimed to evaluate chronic kidney disease (CKD) epidemiology in a large cohort of Fabry disease patients, with a particular focus on identifying factors associated with early progression to  CKD.

## Methods

### Study design and population

We conducted a prospective observational cohort study with data collected retrospectively from 1st November, 2000 up to and including 1st February, 2022, in a single tertiary centre for metabolic disease in Northwest England. Adults aged 18 years or over with a confirmed genetic diagnosis of Fabry disease were included. Participants with at least three measured serum biochemistry values over a minimum follow-up period of one year or more were included in the renal sub-group analysis. Individuals established on kidney replacement therapy (KRT) prior to the start of the study were excluded.

The study had institutional (study reference: S19MET08-S) and the Health Research Authority (identification number: 262706, reference: 19/HRA/5221) approval, and was exempt from review by a regional ethics committee.

### Data gathering

Primary study outcomes include the development of ESKD requiring initiation of KRT, annual rate of progression of estimated glomerular filtration rate (eGFR), change in urine albumin-to-creatinine ratio (uACR) and non-fatal cardiovascular events. The secondary outcome was all-cause mortality.

Baseline data were defined as collected within 180 days of first clinic appointment; consisting of patient demographics (age, reported gender and ethnicity), past medical history (documented co-morbidities including previous non-fatal cardiovascular events, hypertension, diabetes mellitus, CKD), prior initiation of KRT (defined as haemodialysis, peritoneal dialysis, or kidney transplant)). Hypertension was defined as > 140 mmHg systolic blood pressure and/or diastolic blood pressure of > 90 mmHg, or receiving one or more antihypertensive agents. Fabry disease mutation type, date of diagnosis, medication use, clinical observations including blood pressure, laboratory data (eGFR (calculated using the CKD epidemiology collaboration (CKD-EPI) equation), serum creatinine, urea, corrected calcium (cCa^++^), phosphate, lipid profile, haemoglobin, alpha galactosidase activity, plasma lysosomal Gb3,and uACR were also included. eGFR and uACR criteria were used to define CKD in accordance with established Kidney Disease Improving Global Outcomes (KDIGO) guidelines[9].

### Statistical analysis

Initial baseline data analysis was performed by stratifying data according to sex. Continuous variables were described with medians and interquartile range (IQR), categorical data were expressed as counts and percentages. We used Chi-square testing for categorical covariates and Mann–Whitney *U* test for continuous data. Linear regression analysis was used to calculate the annual rate of change (delta; Δ) of eGFR and uACR using all available values between study baseline and endpoints. Participants were further categorised according to the rate of eGFR progression (‘slow or natural progressors’ defined as eGFR slope ≤ −1 ml/min/year; ‘intermediate progressors’ defined as between −1 and −3 ml/min/year; and ‘fast progressors’ defined as more than −3 ml/min/year). Univariate and subsequent multivariate logistic regression were used to derive significant covariates associated with faster progression of eGFR decline. Additional outcome analysis was stratified to the rate of eGFR progression and those who went on to require KRT with a > 50% drop in eGFR. A *p-*value of < 0.05 was considered statistically significant. All statistical analyses were performed using IBM SPSS Version 24.

## Results

### Baseline characteristics

Four hundred and fourteen Fabry patients were eligible for study inclusion. Of these, nine had incomplete datasets thus were excluded (Suppl. Figure 1) A further ten patients who were already established on KRT before the start of the study were also excluded. Minimum duration of follow-up was set at 180 days after initiation of enzyme replacement therapy/oral chaperone therapy in the disease modifying treatment subgroup, or after initial clinic appointment in the treatment-naïve patient cohort. Two hundred forty-nine participants had biochemical data available for further analysis of renal outcomes, including eGFR and uACR values, for a minimum follow-up period of 1 year. A total of 405 participants had available data meeting eligibility criteria. Of these, 395 participants underwent outcome analysis.

Table 1 presents a summary of baseline participant characteristics detailing demographic and clinical factors, stratified by sex. The median age (IQR) of the full cohort was 42 years (31–55), with most participants identifying as White British (*n* = 398, 98.3%, *p* = 0.38). One hundred twelve (27.7%; female = 62, male = 50) individuals had the classical form of Fabry disease and 178 (44%, female = 90, male = 88) had a non-classical variant, with genetic data being unavailable for 115 (28.4%) participants (Table 1).

**Table 1:** Distribution of baseline study participant characteristics

Baseline variable	Total (*n* = 405)	Female (*n* = 223)	Male (*n* = 182)	*p* value
Age, years	42 (31–55)	39 (27–54)	44 (33–55)	0.066
Ethnicity, White British	398 (98.3%)	218 (97.8%)	180 (98.9%)	0.380
Heart rate, beats per min	65 (57–73)	67 (60–75)	62 (55–71)	** < 0.001**
Systolic BP, mm Hg	126 (115–140)	123 (115–136)	129 (117–143)	**0.006**
Diastolic BP, mm Hg	76 (70–84)	76 (70–84)	77 (70–84)	0.897
BMI, kg/m^2^	26 (23–30)	26 (23–30)	25.7 (22.7–29)	0.214
Smoking	73 (18%)	36 (16.1%)	37 (20.3%)	0.276
Hypertension	81 (20%)	39 (17.5%)	42 (23.1%)	0.162
Diabetes mellitus	12 (3%)	4 (1.8%)	8 (4.4%)	0.125
Hypercholesterolemia	34 (8.4%)	17 (7.6%)	17 (7.6%)	0.535
Cerebrovascular accident	20 (4.9%)	6 (2.7%)	14 (7.7%)	**0.021**
Transient ischaemic attack	8 (2%)	6 (2.7%)	2 (1.1%)	0.252
Ischaemic heart disease	19 (4.7%)	9 (4%)	10 (5.5%)	0.490
Atrial fibrillation	26 (6.4%)	10 (4.5%)	16 (8.8%)	0.079
Asthma	38 (9.4%)	23 (10.3%)	15 (8.2%)	0.477
COPD	4 (1%)	3 (1.3%)	1 (0.5%)	0.420
Kidney Transplant	4 (1%)	0	4 (2.2%)	**0.026**
Genetic variants				
Classical	112 (27.7%)	62 (27.8%)	50 (27.5%)	
Late-onset	178 (44%)	90 (40.4%)	88 (48.4%)	0.171
Not available	115 (28.4%)	71 (31.8%)	44 (24.2%)	
Laboratory values				
Albumin, g/L (*n* = 258)	45 (44–47)	45 (44–47)	46 (43–47)	0.647
cCa^++^, mmol/L (*n* = 288)	2.3 (2.2–2.39)	2.31 (2.22–2.39)	2.3 (2.2–2.37)	0.131
Phosphate, mmol/L (*n* = 288)	1.11 (0.98–1.22)	1.12 (0.98–1.22)	1.10 (0.98–1.23)	0.604
Haemoglobin, g/L (*n* = 304)	137 (130–147)	134 (126–140)	145 (134–151)	** < 0.001**
Creatinine, μmol/L (*n* = 395)	70 (60–85)	62 (57–71)	83 (73–96)	** < 0.001**
eGFR, ml/min/1.73m^2^ (*n* = 395)	104 (87–118)	109 (92–120)	100 (82–113)	**0.001**
uACR, mg/mmol (*n* = 345)	2.6 (0.97–22.2)	1.86 (0.84–10.8)	4.7 (1.28–39.9)	**0.004**
Total Cholesterol, mmol/L (*n* = 278)	4.7 (4–5.5)	4.95 (4.1–5.7)	4.4 (3.75–5.35)	** < 0.001**
HDL, mmol/L (*n *= 278)	1.5 (1.24–1.8)	1.63 (1.39–19.5)	1.35 (1.12–1.6)	** < 0.001**
Lysosomal GB3, nmol/L (*n* = 157)	4.6 (1.9–10.6)	2.5 (1.2–6.6)	7.4 (4.2–19.2)	** < 0.001**
Alfa galactosidase A levels, nmol/ml/hr (*n* = 230)	0.6 (0.3–1.7)	2.3 (1.6–4.3)	0.4 (0.2–0.6)	** < 0.001**

Categorical values are expressed as count and percentage (%), continuous variables are expressed as median (interquartile range, IQR, Q1–Q3). *p*-values calculated by Chi-square and Mann–Whitney *U* tests, respectively. *BP* Blood pressure, *BMI* Body mass index, *COPD*, Chronic obstructive pulmonary disease, *cCa*^*++*^ corrected calcium, *uACR* urine albumin-to-creatinine ratio, *HDL* High density lipoprotein. *p* < 0.05 in bold taken as statistically significant

The median heart rate for the entire cohort was 65 beats per minute (bpm), with women having a slightly higher rate of 67 bpm compared to men (62 bpm, *p* < 0.001). The median systolic blood pressure was 126 mmHg, this being 123mmHg in women, whilst men had a median of 129 mmHg (*p* = 0.006). Approximately 18% of all participants were smokers, with a greater prevalence among men (20.3%) compared with women (16.1%). The median body mass index (BMI) was 26 kg/m^2^ for the total study cohort, with no significant difference between women and men (*p* = 0.214).

Two hundred forty-nine participants (63%) had a diagnosis of CKD at the time of study entry. Among them, 222 had an eGFR value > 60; 27 subjects had an eGFR < 60, and 92 individuals had a baseline uACR > 3 mg/mmol with an eGFR reading of > 60 (Suppl.Tables 2 & 3).

Hypertension was present in 20% (*n* = 81) of the total study population, with a lower prevalence in women (17.5%, *n* = 39 vs 23.1%, *n* = 42 in men), but this was not of statistical significance (*p* = 0.162). The presence of diabetes mellitus (observed in 3%, *n* = 12), and hypercholesterolemia (present in 8.4% of the cohort) had similar prevalence in both sexes. A history of cerebrovascular accident (CVA; stroke) was reported by 4.9% (*n* = 20) of total participants, with men having a higher prevalence (7.7%, *n* = 14) compared with women (2.7%, *n* = 6; *p* = 0.021). Transient ischaemic attacks were documented in 2% of the participants, with no significant differences between sexes. Ischaemic heart disease was present in 4.7% (*n* = 19) of the study population and atrial fibrillation was noted in 6.4% of participants, again with no significant inter-sex differences.

Men had lower eGFR (100 (82–113) vs 109 (92–120) ml/min/1.73m^2^, *p* = 0.001), serum total cholesterol (4.4 (3.75–5.35) vs 4.95 (4.1–5.7) mmol/L, *p* < 0.001) and serum HDL cholesterol (1.35 (1.12–1.6) vs 1.63 (1.39–19.5) mmol/L, *p* < 0.001) than women, respectively (Table 1). Analysis of laboratory blood samples of participants revealed men had higher haemoglobin concentration (145 (IQR: 134–151) g/L vs 134 (126–140) g/L, *p* < 0.001), serum creatinine (83 (73–96) μmol/L vs 62 (57–71) μmol/L, *p* < 0.001), uACR (4.7 (1.28–39.9) mg/mmol vs 1.86 (0.84–10.8) mg/mmol, *p* = 0.004), and lysosomal GB3 (7.4 ng/ml (IQR:4.2–19.2) vs 2.5 ng/ml (IQR:1.2–6.6)) than women, respectively. Men had lower the serum alpha galactosidase levels at baseline  (0.4 (0.2–0.6) vs 2.3 (1.6–4.3) nmol/ml/hr, *p* < 0.001) (Table 1). Almost all medications, including antihypertensive agents and statins, were prescribed more frequently in men than women (Suppl.Table 1).

## Incident outcomes: treatment initiation, KRT, non-fatal cardiovascular events and all-cause mortality

Table 2 displays incident study outcomes. Over a median of 6.4 years (3.6–12.2), men required disease modifying treatments more frequently than women (85.5%, *n* = 147 vs 41.3%, *n* = 92 in women, *p* < 0.001), progressed to KRT (7%, *n* = 12 vs 0.4%, *n* = 1, *p* < 0.001) and experienced more incident non-fatal cardiovascular events (43.6%, *n* = 75 vs 18.9%, *n* = 42, *p* < 0.001). Male participants also demonstrated a greater rate of all-cause mortality (12.2%, *n* = 21 vs 3.6%, *n* = 8, *p* < 0.001).

**Table 2:** Incident events observed in the study, stratified by sex

Outcome variable	Total (*n* = 395)	Female (*n* = 223)	Male (*n* = 172)	*p* value
DMT	239 (60.5%)	92 (41.3%)	147 (85.5%)	** < 0.001**
KRT	13 (3.3%)	1 (0.4%)	12 (7%)	** < 0.001**
NFCVE	117 (29.7%)	42 (18.9%)	75 (43.6%)	** < 0.001**
All-cause mortality	29 (7.3%)	8 (3.6%)	21 (12.2%)	** < 0.001**
Δ eGFR^†^ (*n* = 260)	−0.60 (−1.99 to −0.04)	−0.49 (−1.6 to−0.05)	−0.83 (−2.1 to −0.03)	0.469
Follow-up, years	6.36 (3.6–12.2)	6.15 (3.59–11.8)	6.95 (4.16–12.6)	0.316

Categorical values are expressed as number and percentage and *p*-value by Chi-square test. Continuous variables are expressed as median (inter quartile range) and *p*-value by Mann–Whitney *U* test. *p* < 0.05 in bold taken as statistically significant. *DMT* Disease modifying treatment (either enzyme replacement or oral chaperone therapy); *eFGR* estimated glomerular filtration rate measured in ml/min/1.73m^2^/year in *n* = 260 subjects; *NFCVE* Non-fatal cardiovascular event including new-onset arrythmias, permanent pacemaker insertion, myocardial infarction, stroke, heart failure; *KRT* Kidney replacement therapy

Furthermore, Kaplan–Meier survival analysis showed women achieving better survival for all-cause mortality (Log-rank *p* < 0.001) and remaining KRT-free (Log-rank *p* = 0.002; Suppl. Figure 2).

### Subgroup analysis: rate of eGFR progression

We conducted a subgroup analysis on *n* = 260 participants with sufficient kidney biochemical data; at least three values for eGFR and uACR each, over a minimum follow-up period of at least one year (Suppl. Table 2). Rate of progression of eGFR decline was categorised into three groups of ‘slow or natural’, ‘intermediate’ and ‘fast’ progressors (see Methods). Rapid progression of eGFR was significantly associated with advanced age, presence of ischaemic heart disease and transient ischaemic attack, and higher uACR levels when compared with slow progressors over a median follow up of nine years (IQR: 4–13). A higher proportion (42.5%) of patients in the rapid progressor group received a renin angiotensin-aldosterone system inhibitor (RAASi), as indicated. Faster eGFR progression was significantly associated with increased all-cause mortality (17.5%, *n* = 7 vs 5.2%, *n* = 8 in slow progressors, *p* = 0.010). ‘Fast’ eGFR progressors had a higher proportion of individuals whose kidney function declined to the point of requiring KRT, with a sustained decrease in eGFR of 50% or more (25%, *n* = 10 vs 3.9% *n* = 6, *p* < 0.001; Suppl. Table 2 & Suppl. Figure 3).

Table 3 displays results of logistic regression analysis of factors found to be associated with rapid eGFR progression. Multivariate logistic regression after covariate adjustment showed both higher age and higher uACR readings to be significantly associated with rapid eGFR decline (OR: 1.09, 95% CI 1.05–1.14, *p* < 0.001 and OR: 1.01, 95% CI 1.01–1.02, *p* = 0.014 respectively).

**Table 3:** Logistic regression analysis of factors associated with rapid eGFR decline (‘slow or natural’ vs ‘fast’ progressors; *n* = 193)

Variable	Univariate model		Multivariate model	
	OR (95% CI)	*p* value	OR (95% CI)	*p* value
Gender, Male	1.33 (0.66–2.67)	0.427		
Age	1.07 (1.04–1.11)	** < 0.001**	1.09 (1.05–1.14)	** < 0.001**
Systolic BP	1.01 (0.98–1.02)	0.559		
Diastolic BP	0.99 (0.96–1.02)	0.707		
Hypertension	1.16 (0.72–3.4)	0.264		
Diabetes Mellitus	3.02 (0.65–14.1)	0.159		
uACR	1.01 (1–1.01)	**0.014**	1.01 (1.01–1.02)	**0.014**
eGFR	0.98 (0.96–0.98)	** < 0.001**	1.01 (0.98–1.03)	0.548
Alfa galactosidase level	0.94 (0.68–1.31)	0.751		
RAASi	4.4 (2.1–9.5)	** < 0.001**		
DMT	0.95 (0.46–1.9)	0.904		

Multivariate model includes factors that were significant in univariate model by priori

*CI* Confidence interval, *OR* Odds ratio, *uACR* urine albumin-to-creatinine ratio, *BP* Blood pressure, *DMT* Disease-modifying therapy (including enzyme replacement or oral chaperone), *RAASi* Renin–angiotensin–aldosterone system inhibitors. *p* < 0.05 in bold taken as statistically significant

### Subgroup analysis: rate of uACR progression

Within the same sub-cohort of participants, we also undertook analysis with quantified uACR measurements (Table 4). When compared to those in the lowest uACR group (< 3 mg/mmol), individuals with higher uACR (> 30 mg/mmol) were more likely to be male (65.3%, *n* = 32 vs 36.3%, *n* = 49, *p* = 0.001), be older (49 vs 44 years, *p* = 0.02), have higher systolic blood pressure (138 vs 126 mmHg, *p* < 0.001), greater RAASi use (44.9%, *n* = 22 vs 13.3%, *n* = 18, *p* < 0.001) and more likely to be receiving disease modifying treatments (89.8%, *n* = 44 vs 57.8%, *n* = 78, *p* < 0.001) over a median of 9 years. Moreover, those with uACR > 30mg/mmol experienced higher rates of non-fatal cardiovascular events (55.1%, *n* = 27 vs 31.1%, *n* = 42, *p* = 0.006), all-cause mortality (18.4%, *n *= 9 vs 4.4%, *n* = 6, *p* = 0.007) and proceeded to KRT (22.4%, *n* = 11 vs 0, *p* < 0.001).

**Table 4:** Sub cohort participant characteristics and outcomes according to uACR (*n* = 249)

Baseline variable (*n* = 249)	uACR < 3 mg/mmol (*n* = 135)	uACR 3-30 mg/mmol (n = 65)	uACR > 30 mg/mmol (*n* = 49)	*p* value
Gender, Male	49 (36.3%)	33 (50.8%)	32 (65.3%)	**0.001**
Age, years	44 (33–55)	50 (40–60)	49 (41–57)	**0.020**
Systolic BP, mmHg	126 (115–137)	128 (116–147)	138 (122–155)	** < 0.001**
Diastolic BP, mmHg	77 (77–84)	77 (72–85)	81 (74–88)	0.088
RAASi	18 (13.3%)	15 (23.1%)	22 (44.9%)	** < 0.001**
DMT	78 (57.8%)	48 (73.8%)	44 (89.8%)	** < 0.001**
Outcome variable				
Δ uACR, (mg/mmol/year)	−0.001 (−0.03 to 0.027)	0.03 (−0.12 to 0.18)	0.28 (−0.57 to1.18)	0.056
Δ eGFR, (ml/min/1.73m^2^/year)	−0.44 (−1.51 to −0.03)	−0.62 (−2.1 to -0.08)	−1.35 (−3.7 to −0.09)	0.080
NFCVE	42 (31.1%)	30 (46.2%)	27 (55.1%)	**0.006**
All-cause mortality	6 (4.4%)	9 (13.8%)	9 (18.4%)	**0.007**
KRT	0	1 (1.5%)	11 (22.4%)	** < 0.001**

Categorical values expressed as number and percentage and *p*-value by Chi-square test. Continuous variables are expressed as median (inter-quartile range) and *p*-value by Kruskal–Wallis H test

*BP* Blood pressure, *DMT* Disease-modifying therapy (including enzyme replacement or oral chaperone), *eGFR* estimated glomerular filtration rate, *NFCVE* Non-fatal cardiovascular events, *RAASi* Renin–angiotensin–aldosterone system inhibitors, *KRT* Kidney replacement therapy, *uACR* urine albumin-to-creatinine ratio. *p* < 0.05 in bold considered statistically significant

### Subgroup analysis: baseline CKD status

We undertook additional analyses of 249 participants based on those who had CKD at baseline according to eGFR and uACR criteria (Suppl.Table 3). Of these, 222 were categorised as ‘early CKD’, i.e. classified as G2A3 or less. Those with early CKD at baseline were older (> 55 vs 51 years, *p* < 0.001), reported frequent RAASi use (26 vs 17 patients, *p* = 0.002), were receiving disease modifying treatments, and experienced a greater rate of incident non-fatal cardiovascular events (45 vs 39, *p* = 0.008) compared with those with no CKD at baseline, respectively (Suppl.Table 4). A higher proportion of patients with a classical genetic variant developed CKD at follow-up compared to the late onset variant (41.7% vs 20.6%, *p* = 0.043) (Suppl.Table 5).

Furthermore, we evaluated 130 individuals who were CKD-free at study initiation but went on to develop incident CKD at follow-up (*n* = 33). Univariate logistical regression of these individuals revealed that increased uACR (OR: 2.65, 95%CI 1.53–4.61, *p* = 0.001) and lower eGFR values (OR: 0.92, 0.94–0.99, *p* = 0.015) are significant risk factors, despite being within ‘normal’ clinical ranges (Suppl.Table 6).

## Discussion

Our study demonstrates that men with Fabry disease are more likely to progress to incident renal decline over at least six years, despite no significant inter-sex differences with respect to traditional cardiovascular and/or renal risk factors (Table 1). Out of 395 participants with Fabry disease, approximately 33% were CKD-free at baseline. Further analysis of 260 participants showed that half of this cohort did not have CKD at entry into the study, with approximately 25% going on to develop incident CKD over a follow-up of > 11 years. Overall, men had significantly higher incidence rates of initiating disease modifying treatments, non-fatal cardiovascular events and all-cause mortality, as well as a greater rate of decline in eGFR and progression to KRT. Further sub-cohort analysis revealed that advanced age, higher uACR (> 2.3 mg/mol), and history of ischaemic heart disease and/or transient ischaemic attack were associated with faster eGFR progression, irrespective of sex. Moreover, those going on to develop incident CKD were on average eight years older, had pre-existing ischaemic heart disease, and experienced a higher rate of eGFR progression than those who remained CKD-free at the end of follow-up. It is worth noting that our study accounts for both age-related physiological decline (‘slow or natural progressors’ in eGFR in addition to including ‘intermediate’ and ‘fast progressors’, which is above and beyond the acceptable age-related decline of approximately −1 ml/min/year) [10]. We acknowledge that another key factor influencing eGFR progression is the timely start of disease modifying treatment. Whilst our database records the start time of these therapies, it does not always confirm the timeliness of their initiation in terms of clinical disease activity, as this can be influenced by the referral process to our specialist Tertiary centre. It is well-documented in the literature that the interval between disease presentation and diagnosis can span 7 to 10 years, which we have captured in our follow-up duration.

A further important finding from this study is that individuals exhibiting elevated uACR or decreased eGFR, *even when these values remain within clinically accepted ‘normal’ ranges, are at increased risk of developing CKD*. This observation underscores the importance of vigilant monitoring of these biomarkers. For instance, a subtle increase in uACR from 1 to 2 mg/mmol, or a decline in eGFR from 89 to 80 ml/min/1.73m^2^, should prompt suspicion of potential CKD progression and might necessitate early intervention, including the initiation of disease-modifying treatments. Such proactive management strategies could potentially mitigate the progression to overt CKD.

It has been previously shown that a urine protein-to-creatinine ratio (uPCR) of > 1000 mg/g (> 100 mg/mmol) in Fabry disease significantly increased the risk of developing ESKD, irrespective of whether patients received enzyme replacement therapy [11]. We further characterised baseline participant demographics and outcome measures according to quantified uACR cut-offs, demonstrating that individuals with uACR > 30 mg/mmol were also more likely to experience outcomes of non-fatal cardiovascular events, all-cause mortality, and require KRT.

Our study indicates that even small increases in uACR may predict renal decline in those developing CKD. Building on our findings, there may be a role for the initiation of newer drugs used in renal care, such as Sodium-glucose co-transporter-2 (SGLT2) inhibitors; upon detecting proteinuria in Fabry patients there is a case for early intervention with SGLT2 inhibitors in order to slow eGFR progression. The ongoing DEFY study could provide insights into the use of SGLT2 inhibitors in Fabry treatment protocols [12].

We show that uACR > 30 mg/mmol is associated with a statistically significant increased risk of developing ESKD, thus confirming the importance of regular uACR testing as part of Fabry clinical care. Interestingly, age was an independent risk factor for rapid rate of eGFR decline, whilst other established risk factors for developing CKD such as hypertension and diabetes were not found to be of significance in this study. This is at odds with a paper published by Nasu et al. which found elevated systolic blood pressure was associated with a faster decline in eGFR in patients with Fabry disease [13]. We acknowledge this is likely due to the relatively low number of patients with hypertension (22.9%) and diabetes (2.7%) at baseline, who would then continue to develop renal sequelae from these conditions, irrespective of having Fabry disease.

Participants taking RAAS inhibitors in our study were shown to have reduced rates of eGFR decline; this is in keeping with published data of patients with proteinuria, in whom these drugs have long demonstrated a renoprotective effect [14]. A higher proportion (42.5%) of patients in the rapid progressor group received a RAASi, as indicated. The main European Fabry Working Group recommendation is timely initiation of disease modifying treatment in all patients with confirmed Fabry disease who demonstrate any level of albuminuria, or who have kidney failure, and this has been incorporated into the UK Fabry guidelines [15]. Given that detectable levels of albuminuria and reduced eGFR are associated with faster decline in eGFR and worse outcomes, there is a case for the initiation of disease modifying treatments in Fabry patients who are at higher risk of developing kidney dysfunction.

Evaluating this study within the wider context, our findings agree with the current literature, and show a consistent correlation between declining eGFR and adverse cardiovascular and renal events in Fabry disease [16–20]. Indeed, men, who typically represent the classical and severe phenotype, have greater uACR at baseline (4.7 v 1.86 mg/mmol) and lower eGFR values (100 v 109) than women. During follow-up, male subjects also exhibit a greater decline in eGFR. Furthermore, those who have a faster progression in decline of eGFR also go on to require KRT, with a higher prevalence of Ischaemic heart disease and increased all-cause mortality.

This study is one of the largest cohort studies of Fabry patients that is inclusive of genetic data, medications, baseline co-morbidities and renal outcomes over ten years of follow-up. However, we acknowledge that Fabry disease is a multi-ethnic condition, and a key limitation of our study was that most of our cohort were of White British ethnicity. This study was conducted in accordance with the ethical standards of the institutional committee and with the 1964 Helsinki Declaration and its later amendments. Informed consent was obtained from all individual participants included in the study. No animal studies were conducted as part of this research. Our data encompass more than 20 years, during which treatments have evolved and been modified according to patient response. Consequently, we were unable to distinguish differences in outcomes based on individual disease-modifying therapies. Moreover, smaller subgroup sample sizes render it challenging to draw definitive conclusions to change clinical practice and guidelines; thus, there is scope for future work incorporating larger, multi-ethnic populations.

In conclusion, our study validates the existing associations between declining eGFR and adverse renal outcomes within Fabry patients, above and beyond that of age-related decline. We have additionally identified uACR as an early and sensitive predictive biomarker of adverse renal outcomes, with those approaching near ‘normal’ values raising suspicion for increased clinical monitoring and possible therapeutic intervention prior to the onset of CKD.

## Supplementary Information

Below is the link to the electronic supplementary material.

Supplementary file1 (DOCX 98 KB)

## Data Availability

The data underlying this article will be shared on reasonable request to the corresponding author.

## References

[1] Arends M et al (2017) Characterization of classical and nonclassical Fabry disease: a multicenter study. J Am Soc Nephrol 28(5):1631–1641 27979989 10.1681/ASN.2016090964 PMC5407735

[2] Germain DP (2010) Fabry disease. Orphanet J Rare Dis 5:30 21092187 10.1186/1750-1172-5-30 PMC3009617

[3] Rajan JN et al (2021) Review of mechanisms, pharmacological management, psychosocial implications, and holistic treatment of pain in fabry disease. J Clin Med. 10.3390/jcm10184168 34575277 10.3390/jcm10184168 PMC8472766

[4] Zarate YA, Hopkin RJ (2008) Fabry’s disease. Lancet 372(9647):1427–1435 18940466 10.1016/S0140-6736(08)61589-5

[5] Di Risi T et al (2021) DNA methylation impact on Fabry disease. Clin Epigenetics 13(1):24 33531072 10.1186/s13148-021-01019-3 PMC7852133

[6] Meikle PJ et al (1999) Prevalence of lysosomal storage disorders. JAMA 281(3):249–254 9918480 10.1001/jama.281.3.249

[7] Gilchrist M et al (2023) Prevalence of Fabry disease-causing variants in the UK Biobank. J Med Genet 60(4):391–396 35977816 10.1136/jmg-2022-108523 PMC10086508

[8] Perretta F, Jaurretche S (2023) Fabry disease: switch from enzyme replacement therapy to oral chaperone migalastat: what do we know today? Healthcare (Basel). 10.3390/healthcare11040449 36832983 10.3390/healthcare11040449 PMC9957019

[9] Chapter 1: Definition and classification of CKD*.* Kidney Int Suppl (2011), 2013. **3**(1): p. 19–62. 10.1038/kisup.2012.64 PMC4089693 25018975

[10] Cohen E, Nardi Y, Krause I, Goldberg E, Milo G, Garty M, Krause I(2014) A longitudinal assessment of the natural rate of decline in renal function with age. J Nephrol 27(6):635–41 24643437 10.1007/s40620-014-0077-9

[11] Warnock DG et al (2012) Renal outcomes of agalsidase beta treatment for Fabry disease: role of proteinuria and timing of treatment initiation. Nephrol Dial Transpl 27(3):1042–1049 10.1093/ndt/gfr420 PMC3289896 21804088

[12] Battaglia Y et al (2023) Dapaglifozin on albuminuria in chronic kidney disease patients with FabrY disease: The DEFY study design and protocol. J Clin Med. 10.3390/jcm12113689 37297884 10.3390/jcm12113689 PMC10253838

[13] Nasu M et al (2023) A nationwide cross-sectional analysis of biopsy-proven Fabry nephropathy: the Japan Renal Biopsy Registry. Clin Exp Nephrol 27(2):141–150 36329296 10.1007/s10157-022-02287-w PMC9845163

[14] Torra R (2008) Renal manifestations in Fabry disease and therapeutic options. Kidney Int Suppl 111:S29-32 10.1038/ki.2008.522 19039306

[15] Biegstraaten M et al (2015) Recommendations for initiation and cessation of enzyme replacement therapy in patients with Fabry disease: the European Fabry Working Group consensus document. Orphanet J Rare Dis 10:36 25885911 10.1186/s13023-015-0253-6 PMC4383065

[16] Spinelli L et al (2010) Does left ventricular function predict cardiac outcome in Anderson-Fabry disease? Int J Cardiol 145(1):24–28. 10.1016/j.ijcard.2009.03.099 10.1007/s10554-020-02105-y PMC8026432 33211238

[17] Feriozzi S et al (2015) Effects of baseline left ventricular hypertrophy and decreased renal function on cardiovascular and renal outcomes in patients with Fabry disease treated with agalsidase alfa: a Fabry outcome survey study. Clin Ther 37(10):2484–2498. 10.1016/j.clinthera.2015.09.013 10.1016/j.clinthera.2020.10.007 33218740

[18] Lenders M et al (2017) Renal function predicts long-term outcome on enzyme replacement therapy in patients with Fabry disease. Nephrol Dial Transplant 32(12):2090–2098. 10.1093/ndt/gfw487 27679524 10.1093/ndt/gfw334

[19] Patel MR et al (2011) Cardiovascular events in patients with Fabry disease: natural history data from the Fabry Registry. Am J Cardiol 108(1): 99–105. 10.1016/j.amjcard.2011.02.340 21349401 10.1016/j.jacc.2010.11.018

[20] Arends M et al (2017) Retrospective study of long-term outcomes of enzyme replacement therapy in Fabry disease: analysis of prognostic factors. PLoS One 12(8):e0182379. 10.1371/journal.pone.0182379 28763515 10.1371/journal.pone.0182379 PMC5538714

