# Supplementary material for: Epidemiology and early predictors of Fabry nephropathy: evaluation of long-term outcomes from a national Fabry centre
Source: J Nephrol. 2024 Dec 19;38(2):579–87. doi: 10.1007/s40620-024-02170-9 (PMC11961510; doi:10.1007/s40620-024-02170-9)
Supplement: Supplementary file 1 — Supplementary file1 (DOCX 98 KB) [file 40620_2024_2170_MOESM1_ESM.docx]

**Supplementary Materials**

**Supplementary Figure 1.** Flowchart of patients included in the study.

**Supplementary Figure 2.** Kaplan-Meier curves of cumulative survival from all-cause mortality (A) and RRT-free survival (B) respectively in 395 patients stratified to sex.

**A)**


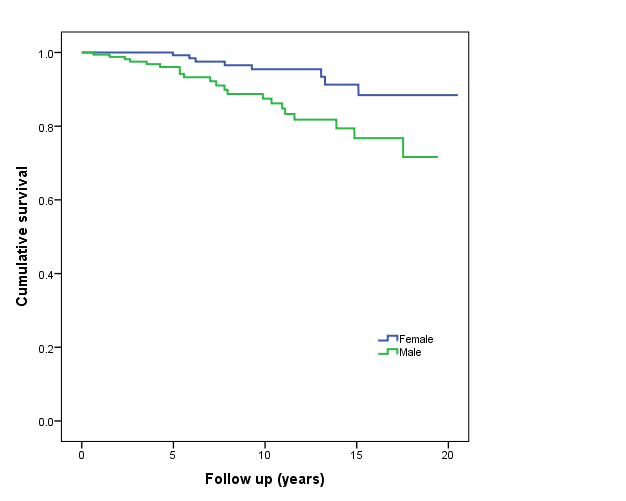


**B)**


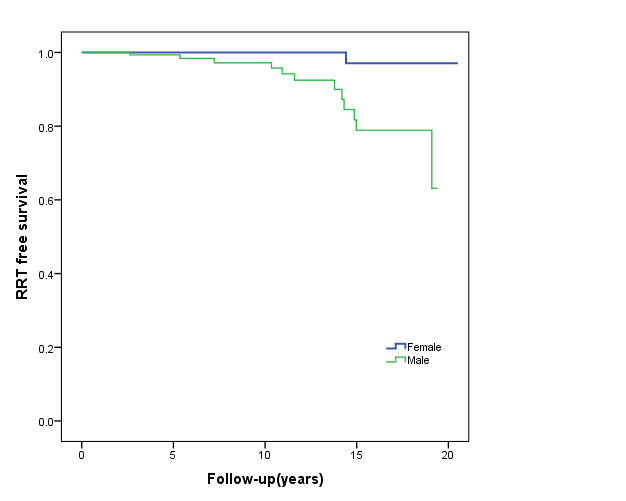


**Supplementary Figure 3.** Kaplan-Meier curves of cumulative RRT-free survival and with a sustained 50% reduction in eGFR) stratified to progression status.


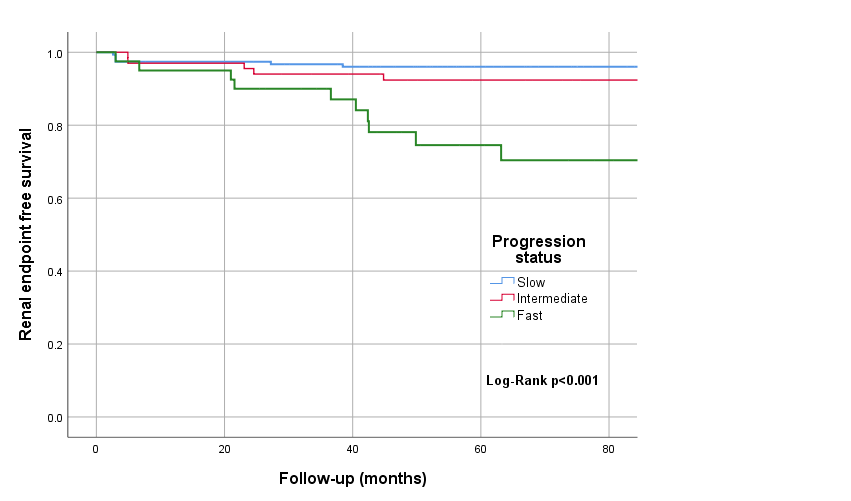


**Supplementary Table 1.** Distribution of baseline study participant medications.

| **Variable** | **Total**  **(n = 405)** | **Female**  **(n = 223)** | **Male**  **(n = 182)** | **p-value** |
| --- | --- | --- | --- | --- |
| ACEi | 57 (14.1%) | 21 (9.4%) | 36 (19.8%) | **0.003** |
| ARB | 17 (4.2%) | 6 (2.7%) | 11 (6%) | 0.094 |
| Beta blockers | 70 (17.3%) | 20 (9%) | 50 (27.5%) | **<0.001** |
| MRA | 3 (0.7%) | 0 | 3 (1.6%) | 0.054 |
| Loop diuretics | 8 (2%) | 2 (0.9%) | 6 (3.3%) | 0.084 |
| Thiazide diuretics | 12 (3%) | 9 (4%) | 3 (1.6%) | 0.159 |
| Calcium channel blocker (BP) | 25 (6.2%) | 9 (4%) | 16 (8.8%) | **0.048** |
| Calcium channel blocker (rate control) | 9 (2.2%) | 4 (1.8%) | 5 (2.7%) | 0.517 |
| Nitrates | 13 (3.2%) | 4 (1.8%) | 9 (4.9%) | 0.073 |
| Aspirin | 54 (13.3%) | 18 (8.1%) | 16 (19.8%) | **0.001** |
| Clopidogrel | 13 (3.2%) | 9 (4%) | 4 (2.2%) | 0.297 |
| Statin | 75 (18.5%) | 25 (11.2%) | 50 (27.5%) | **<0.001** |
| Metformin | 8 (2%) | 2 (0.9%) | 6 (3.3%) | 0.084 |
| Warfarin | 17 (4.2%) | 7 (3.1%) | 10 (5.5%) | 0.240 |
| NOAC | 6 (1.55) | 1 (0.4%) | 5 (2.7%) | 0.057 |
| Digoxin | 3 (0.7%) | 2 (0.9%) | 1 (0.5%) | 0.685 |

Categorical values expressed as count and percentage (%), P-values calculated by Chi-square test. ACEi, angiotensin-converting enzyme inhibitor; ARB, angiotensin-receptor blocker; BP, blood pressure; MRA, mineralocorticoid receptor antagonists; NOAC, novel oral anticoagulant. P <0.05 taken as statistically significant.

| **Baseline variable** | **Total (260)** | **Slow or natural** ≤ -1 ml/min/year **(n = 153)** | **Intermediate** between -1 and -3 ml/min/year  **(n = 67)** | **Fast** > -3 ml/min/year  **(n = 40)** | **P value**  (slow vs fast) |
| --- | --- | --- | --- | --- | --- |
| Gender, male | 116 (45) | 62 (40.5) | 35 (52.2) | 19 (47.5) | 0.426 |
| Age, years | 45 (35-57) | 41 (32-53) | 49 (39-58) | 55 (47-63) | **<0.001** |
| Hypertension | 58 (22.3) | 33 (21.6) | 13 (19.4) | 12 (30) | 0.262 |
| Diabetes Mellitus | 7 (2.7) | 4 (2.6) | 0 | 3 (7.5) | 0.141 |
| Hypercholesterolemia | 26 (10) | 14 (9.2) | 6 (9) | 6 (15) | 0.280 |
| IHD | 14 (5.4) | 4 (2.6) | 4 (6) | 6 (15) | **0.002** |
| TIA | 7 (2.7) | 2 (1.3) | 2 (3) | 3 (7.5) | **0.028** |
| Stroke | 13 (5) | 7 (4.6) | 4 (6) | 2 (5) | 0.910 |
| Smoking | 55 (21.2) | 32 (20.9) | 18 (26.9) | 5 (12.5) | 0.352 |
| Systolic BP,mmHg | 128 (116-143) | 129 (116-140) | 125 (116-141) | 129 (115-151) | 0.625 |
| Diastolic BP,mmHg | 77 (72-85) | 78 (72-86) | 76 (72-83) | 77 (68-86) | 0.775 |
| uACR, mg/mmol | 2.64 (0.97-20.3) | 2.3 (0.97-8.4) | 2.07 (0.74-23.2) | 13.5 (1.39-59.6) | **0.002** |
| RAASi | 55 (21.2) | 22 (14.4) | 16 (23.9) | 17 (42.5) | **<0.001** |
| DMT | 178 (68.5) | 101 (66) | 51 (76.1) | 26 (65) | 0.904 |
| **Outcome variable** |  |  |  |  |  |
| RRT | 12 (4.6) | 6 (3.9) | 4 (6) | 2 (5) | 0.761 |
| RRT and >50% drop in eGFR | 21 (8.1) | 6 (3.9) | 5 (7.5) | 10 (25) | **<0.001** |
| NFCVE | 101 (38.8) | 50 (32.7) | 33 (49.3) | 18 (45) | 0.146 |
| All-cause mortality | 24 (9.2) | 8 (5.2) | 9 (13.4) | 7 (17.5) | **0.010** |
| Follow-up, years | 9 (4-13) | 10.5 (5-15) | 10 (6-13) | 5 (3-9) | **<0.001** |

**Supplementary Table 2**. Sub-cohort participant characteristics and outcomes stratified to rate of eGFR progression (n = 260).

Categorical values expressed as number and percentage and p-value by Chi-square test. Continuous variables expressed as median (inter quartile range) and p-value by Mann-Whitney U test. ‘slow or natural progressors’ defined as eGFR slope of ≤ -1 ml/min/year; ‘intermediate progressors’ defined as between -1 and -3 ml/min/year; and ‘fast progressors’; defined as those with an eGFR slope of more than -3 ml/min/year. BP, blood pressure; DMT, disease-modifying therapy (including enzyme replacement or oral chaperone); IHD, ischaemic heart disease, NFCVE, non-fatal cardiovascular events, RAASi, renin-angiotensin-aldosterone system inhibitors; RRT, renal replacement therapy; TIA, transient ischaemic attack. P<0.05 considered statistically significant.

**Supplementary Table 3.** Distribution of participants based on CKD at baseline (n = 249). These participants are on standard treatments including Renin-angiotensin-aldosterone system inhibitors (RAASi) and Fabry disease modifying therapies (enzyme replacement therapy or oral chaperone therapy).

| **Distribution based on CKD Stage at baseline (n = 249)** | **uACR<3 mg/mmol**  **(n = 135)** | **uACR 3-30 mg/mmol**  **(n = 65)** | **uACR>30 mg/mmol**  **(n = 49)** |
| --- | --- | --- | --- |
| **G1** (eGFR >/=90) | 99 (73.3%) | 40 (61.5%) | 17 (14.2%) |
| **G2** (eGFR 60-89) | 31 (23%) | 22 (33.8%) | 13 (26.5%) |
| **G3a** (eGFR 45-59) | 5 (3.7%) | 1 (1.53%) | 7 (14.2%) |
| **G3b** (eGFR 30-44) | 0 | 1 (1.53%) | 8 (16.3%) |
| **G4** (eGFR 15-29) | 0 | 0 | 3 (6.1%) |
| **G5** (eGFR <15) | 0 | 1 | 1 (2.04%) |

Categorical values expressed as number and percentage. CKD, chronic kidney disease; eGFR, estimated glomerular filtration rate in ml/min/1.73m^2^/year; uACR, urine albumin-to-creatinine ratio.

**Supplementary Table 4.** Comparison of salient baseline characteristics and outcomes in the no CKD and early CKD sub-cohorts (n = 222).

| **Baseline variable** | **G1A1 and G2A1  (n = 130)** | **G1A2**  **(n = 40)** | **G2A2**  **(n = 22)** | **G1A3**  **(n = 17)** | **G2A3**  **(n = 13)** | **p-value** |
| --- | --- | --- | --- | --- | --- | --- |
| Age | 51 (42-64) | 55 (48-64) | 64 (54-73) | 63 (55-68) | 56 (54-65) | **<0.001** |
| Gender, male | 48 (36.9%) | 21 (52.5%) | 10 (45.5%) | 9 (52.9%) | 6 (46.2%) | 0.379 |
| RAASi | 17 (13.1%) | 6 (15%) | 8 (36.4%) | 6 (35.3%) | 6 (46.25) | **0.002** |
| DMT | 74 (56.9%) | 28 (70%) | 18 (81.8%) | 14 (82.4%) | 11 (84.6%) | **0.023** |
| **Outcome variable** |  |  |  |  |  |  |
| Δ uACR, mg/mmol/year | 0.01 (-0.007 to 0.025) | 0 (-0.03 to 0.05) | 0.02 (-0.06 to 0.15) | 0.06 (-0.18 to 0.19) | 0.25 (-0.57 to 1.20) | 0.420 |
| Δ eGFR, ml/min/1,73m^2^/year | -0.44 (-1.51 to-0.037) | -0.42 (-1.83 to-0.061) | -1.05 (-2.48 to -0.47) | -2.11 (-4.05 to -0.68) | -1.22 (-4.3 to -0.09) | 0.054 |
| NFCVE | 39 (30%) | 16 (40%) | 11 (50%) | 8 (47.1%) | 10 (76.9%) | **0.008** |
| All-cause mortality | 5 (3.8%) | 4 (10%) | 3 (13.6%) | 3 (17.6%) | 1 (7.7%) | 0.147 |

Categorical values expressed as number and percentage and p-value by Chi-square test. Continuous variables expressed as median (inter quartile range) and p-value by Kruskal-Wallis H test. DMT, disease-modifying therapy (including enzyme replacement or oral chaperone); eGFR, estimated glomerular filtration rate; NFCVE, non-fatal cardiovascular events, RAASi, renin-angiotensin-aldosterone system inhibitors; uACR, urine albumin-to-creatinine ratio. P < 0.05 considered statistically significant.

**Supplementary Table 5.** Distribution of baseline characteristics of the 130 non-CKD group (G1A1 and G2A1) patients based on incident CKD at follow-up.

| **Variable** | **Developed CKD**  **(n = 33)** | **Not developed CKD (n = 97)** | **P value** |
| --- | --- | --- | --- |
| Age, years | 49 (36-61) | 41 (33-53) | 0.148 |
| Gender, male | 15 (45.5%) | 33 (34%) | 0.240 |
| Ethnicity, Caucasian | 32 (97%) | 95 (98%) | 0.406 |
| Heart rate, per min | 61 (58-71) | 65 (57-73) | 0.748 |
| Systolic BP, mm Hg | 125 (117-140) | 126 (115-136) | 0.404 |
| Diastolic BP, mm Hg | 80 (73-86) | 76 (71-83) | 0.121 |
| Body mass index, Kg/m^2^ | 25.3 (22.6-28.7) | 27 (23.7-30.3) | 0.158 |
| Smoking | 7 (21.2%) | 19 (19.6%) | 0.840 |
| Hypertension | 4 (12.1%) | 15 (15.5%) | 0.639 |
| Diabetes mellitus | 0 | 3 (3.1%) | 0.307 |
| Hypercholesterolemia | 1 (3%) | 10 (10.3%) | 0.194 |
| Stroke | 1 (3%) | 4 (4.1%) | 0.778 |
| TIA | 1 (3%) | 1 (1%) | 0.420 |
| IHD | 2 (6.1%) | 0 | **0.015** |
| Atrial fibrillation | 1 (3%) | 3 (3.1%) | 0.986 |
| Asthma | FOS5 (15.2%) | 8 (8.2%) | 0.253 |
| COPD | 1 (3%) | 0 | 0.085 |
| RAASi  Δ eGFR, ml/min/1.73m^2^/year  Δ uACR, mg/mmol/year | 6 (18.2%)  -1.05 (-2.1 to-0.39)  0.53 (0.10 to 0.97) | 11 (11.3%)  -0.3 (-1.15 to 0.06)  - 0.004 (-.0.83 to 1.33) | 0.314  **<0.001**  0.059 |
| Died | 3 (9.1%) | 2 (2.1%) | 0.070 |
| Follow up (years) | 11.1 (6.4-13.7) | 9.2 (4.7-12) | 0.216 |
| Genetic variants, n=92  Classical  Late- onset | 10 (41.7%)  14 (20.6%) | 14 (20.6%)  53 (79.4%) | 0.043 |

Categorical values expressed as number and percentage and p-value by Chi-square test. Continuous variables expressed as median (inter quartile range) and p-value by Kruskal-Wallis H test. DMT, disease-modifying therapy (including enzyme replacement or oral chaperone); eGFR, estimated glomerular filtration rate; NFCVE, non-fatal cardiovascular events, RAASi, renin-angiotensin-aldosterone system inhibitors; uACR, urine albumin: creatinine ratio. P < 0.05 considered statistically significant.

**Supplementary Table 6.** Predictors for developing CKD by logistic regression analysis (130 patients)

| **Variable** | **Univariate** |  |
| --- | --- | --- |
|  | **OR (95% CI)** | **p-value** |
| Gender, Male | 1.61 (0.72-3.6) | 0.242 |
| Age | 1.02 (0.99-1.05) | 0.131 |
| Smoker | 1.11 (0.42-2.92) | 0.840 |
| Systolic BP | 1.01 (0.98-1.03) | 0.431 |
| Diastolic BP | 1.03 (0.99-1.08) | 0.105 |
| Hypertension | 0.75 (0.23-2.45) | 0.640 |
| uACR mg/mmol | 2.65 (1.53-4.61) | **0.001** |
| eGFR ml/min/1.73m^2^ | 0.96 (0.94-0.99) | **0.015** |
| RAASi | 1.73 (0.58-5.14) | 0.381 |
| ERT or Chaperone | 1.03 (0.46-2.30) | 0.930 |

BP, blood pressure in mmHg; eGFR, estimated glomerular filtration rate in ml/min/1.73m^2^/year; ERT, enzyme replacement therapy; RAASi, renin-angiotensin-aldosterone system inhibitors; uACR, urine albumin-to-creatinine ratio.
